# Endotracheal Oxygen Insufflation Associated with Life-Threatening Barotrauma during Apnea Testing

**DOI:** 10.1155/2024/9518817

**Published:** 2024-09-27

**Authors:** Saud Ali Aljasir, Fahad Abdullah Alshammari, Jehan Abdullah Abdul-aziz Fatani, Abdalrhman Al Saadon, Abdulaziz H. Alzeer

**Affiliations:** Department of Critical Care King Khalid University Hospital King Saud University Medical City College of Medicine King Saud University, Riyadh, Saudi Arabia

## Abstract

Apnea testing is a standard method when diagnosing brain or more specifically brainstem death; however, it is imperative to acknowledge the potential for lung injury during this procedure and follow clinical practice guideline recommendations that may reduce the risk of complications. Two cases of barotrauma leading to pneumothorax occurred during apnea tests that used higher-than-recommended oxygen flow rates. Additional data are necessary to clarify the mechanism and incidence of this life-threatening complication.

## 1. Introduction

The apnea test is a critical component of brain death determination that assesses the absence of respiratory drive in response to elevated carbon dioxide levels [1]. Most versions of this test involve temporarily disconnecting the patient from mechanical ventilation, which poses a significant risk of hypoxemia. To mitigate this risk, oxygen insufflation through the endotracheal tube (ETT) is commonly employed to provide oxygen directly to the alveoli and maintain adequate oxygenation levels [2]. Alternatively, employing T-piece systems or continuous positive airway pressure (CPAP) to deliver oxygen via standard corrugated tubing has proven to be as effective as traditional oxygen cannula techniques in maintaining adequate oxygenation during apnea testing [3]. However, this seemingly benign intervention is associated with a risk of severe complications, particularly barotrauma. Numerous case reports describing pneumothorax shortly after initiation of the apnea test suggest more detailed studies are warranted to further understand its incidence and clarify damage mechanisms [4–10]. We add to the literature by reporting two cases of tension pneumothorax that we suspect arose from using a higher-than-recommended oxygen flow rate during ETT insufflation. Our aim in sharing these cases is to increase awareness of the risk of barotrauma associated with apnea testing.

### 1.1. Case 1

**Figure 1:**
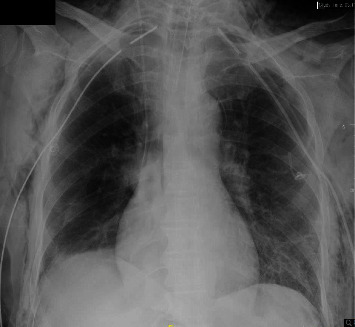
The bilateral subcutaneous emphysema, bilateral intercostal chest drainage tubes, and pneumomediastinum.

A 39-year-old female with no previous medical history arrived at the emergency department unresponsive and pulseless, prompting initiation of cardiopulmonary resuscitation (CPR). Return of spontaneous circulation was achieved following six cycles of defibrillation and medication administration. Medical history obtained from her family revealed that she had fever, cough, shortness of breath, and watery diarrhea one week prior to her admission. A computed tomography (CT) scan of the chest revealed pneumonia with consolidation and ground-glass opacities in both lungs and lower lobe collapse. Additionally, a brain CT scan revealed diffuse cerebral edema without herniation, accompanied by a minor hemorrhagic focus. Except for severe mixed acidosis, her initial laboratory findings were normal. An echocardiogram revealed normal heart function. During the ICU course, the patient remained unresponsive with a Glasgow Coma Scale (GCS) score of 3/15. She required mechanical ventilation on low settings and was intubated with a 7.5 mm ETT. Despite being hemodynamically stable, she exhibited no brain stem reflexes, with fixed and dilated pupils. The patient's neurological status is indicative of a brain stem death, warranting the initiation of the protocol for brain death determination. The apnea test was performed as the final step of the brain death examination. The patient was preoxygenated, and ventilation was set to apnea parameters before disconnection. A 2.5 mm diameter nasal cannula delivering oxygen at 15 L/min was introduced. Two minutes into the apnea test, the patient experienced sudden bilateral pneumothorax with surgical emphysema, prompting immediate termination of the test and insertion of bilateral chest tubes (Figure 1). A subsequent clinical brain death exam confirmed the diagnosis and CT angiogram of the brain showed irreversible damage. The patient's family declined organ donation, and the goal of care was changed to do not resuscitate. The patient passed away peacefully two days later.

### 1.2. Case 2

**Figure 2:**
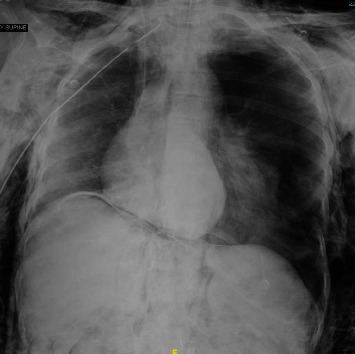
The bilateral subcutaneous emphysema, tension left-sided pneumothorax, pneumoperitoneum, and mediastinal shift to right.

A 54-year-old woman with a history of diabetes, hypertension, and peripheral vascular disease experienced sudden left-sided weakness, numbness, and slurred speech suggestive of a stroke. Initial assessments, including a brain CT scan and angiogram, confirmed an occlusion in the basilar artery. Thrombolysis and mechanical thrombectomy were attempted, but the occlusion persisted, resulting in extensive infarction. The patient's condition deteriorated, leading to intubation with a 7.5 mm ETT and transfer to the ICU. An urgent CT scan showed generalized brain edema, subarachnoid hemorrhage (SAH), cerebellar hemorrhage, and hydrocephalus due to intraventricular hemorrhage. The SAH and hydrocephalus were attributed to the complicated unsuccessful thrombectomy. Complete occlusion of the right common femoral artery due to dissection which led to critical limb ischemia, requiring thrombo-embolectomy. An external ventricular drain was placed to alleviate intracranial pressure. During the ICU course, the patient's neurological status further deteriorated, with a GCS score of 3/15. Despite the absence of brain stem reflexes and cessation of analgosedation after three days, a magnetic resonance imaging brain scan revealed progressive generalized brain edema with herniation. Neurosurgical intervention was not deemed reasonable due to the overall prognosis. During the apnea test, the patient was disconnected from mechanical ventilation, and oxygen insufflation was administered using a 2.5 mm nasal cannula through an ETT at a flow rate of 10 L/min. The patient rapidly developed severe hypoxia and hypotension. The test was aborted, and measures were taken to address extensive surgical emphysema, including bilateral needle decompression and chest tube insertion (Figure 2). Bronchoscopy confirmed intact bronchial airways. A subsequent brain death exam was confirmatory, and a CT angiogram confirmed the absence of blood flow to the brain. Organ donation was declined in accordance with the family's wishes.

## 2. Discussion

Our two cases showcase the life-threatening complication of pneumothorax during endotracheal oxygen insufflation, a procedure aimed at ensuring patient oxygenation during the apnea test, a critical component of the brain death assessment. Recognizing pneumothorax as a potential contributor to hemodynamic or respiratory compromise during apnea testing is crucial for prompt intervention. The American Academy of Neurology guidelines recommend a 6 L/min oxygen flow rate and the strategic placement of the oxygen catheter superior to the carina to ensure both accuracy and safety during the procedure [2]. These guidelines aim to minimize the risks associated with the procedure, such as the complications observed in our cases. The precise mechanism of pneumothorax during apnea testing remains uncertain. One theory suggests it results from direct trauma to the tracheobronchial tree during catheter insertion [5, 7]. Alternatively, endotracheal oxygen insufflation may result in pneumothorax when the oxygen flow rate exceeds the lung's ability to evenly distribute or expel the gas, possibly due to inadequate exhalation [5, 10]. This could be especially relevant when spontaneous respiratory efforts are absent during the apnea test, altering thoracic cavity pressure dynamics. Consequently, localized overdistension due to air trapping may occur, leading to subsequent alveolar rupture that allows air to leak into the pleural space and culminate in bilateral pneumothorax. A systematic review of pneumothorax occurrences during apnea testing found that eight of twelve (67%) patients had oxygen flow rates ≥8 L/min, greater than the 6 L/min recommended in the American Academy of Neurology guidelines. Additionally, if the diameter of the oxygen cannula closely matches that of the ETT, oxygen outflow can be obstructed [11]. A mannequin-based investigation using an oxygen flow rate of 6 L/min reported a notable increase in airway pressures and volumes when the internal cannula-to-ETT ratio exceeded 0.7, leading to a recommendation of a ratio ≤0.7 [11]. Pneumothorax in both of our cases occurred during an oxygen flow rate exceeding 6 L/min, providing further support for the guideline recommendation. This potentially led to localized air trapping, increased alveolar pressure, and subsequent alveolar injury; however, these associations were not verified. Our cases emphasize the importance of vigilant monitoring and prompt intervention for potential barotrauma during the apnea test. Further studies are warranted to elucidate the mechanisms underlying pneumothorax during this procedure to improve patient safety protocols.

## 3. Conclusion

These two cases highlight the rare but critical risk of pneumothorax associated with endotracheal oxygen insufflation during the assessment of brain death. During apnea testing, patient safety and risk mitigation warrant strict adherence to the guidelines on oxygen flow rates and cannula-to-endotracheal tube ratios. Utilizing CPAP or T-piece systems should be considered viable strategies to reduce the risk of complications.

## Data Availability

The data used in this study can be requested from the corresponding author if warranted.
